# Targeting Cancer Stem Cells with Novel 4-(4-Substituted phenyl)-5-(3,4,5-trimethoxy/3,4-dimethoxy)-benzoyl-3,4-dihydropyrimidine-2(1*H*)-one/thiones

**DOI:** 10.3390/molecules21121746

**Published:** 2016-12-19

**Authors:** Mashooq Ahmad Bhat, Abdullah Al-Dhfyan, Mohamed A. Al-Omar

**Affiliations:** 1 Department of Pharmaceutical Chemistry, College of Pharmacy, King Saud University, Riyadh 11451, Saudi Arabia; malomar1@ksu.edu.sa; 2 Stem Cell & Tissue Re-Engineering Program, Research Center, King Faisal Specialized Hospital & Research Center, MBC-03, P.O. Box 3354, Riyadh 11211, Saudi Arabia; harm101696@hotmail.com; 3 Department of Pharmacology and Toxicology, College of Pharmacy, King Saud University, Riyadh 11451, Saudi Arabia

**Keywords:** cancer stem cells, antitumor activity, dihydropyrimidine, enaminones

## Abstract

Novel 4-(4-substituted phenyl)-5-(3,4,5-trimethoxy/3,4-dimethoxy)-benzoyl-3,4-dihydropyrimidine-2(1*H*)-one/thione derivatives (**DHP** **1**–**9**) were designed, synthesized, characterized and evaluated for antitumor activity against cancer stem cells. The compounds were synthesized in one pot. Enaminones **E1** and **E2** were reacted with substituted benzaldehydes and urea/thiourea in the presence of glacial acetic acid. The synthesized compounds were characterized by spectral analysis. The compounds were screened in vitro against colon cancer cell line (LOVO) colon cancer stem cells. Most of the compounds were found to be active against side population cancer stem cells with an inhibition of >50% at a 10 μM concentration. Compounds **DHP-1**, **DHP-7** and **DHP-9** were found to be inactive. Compound **DHP-5** exhibited an in vitro anti-proliferative effect and arrested cancer cells at the Gap 2 phase (G2) checkpoint and demonstrated an inhibitory effect on tumor growth for a LOVO xenograft in a nude mouse experiment.

## 1. Introduction

Compounds, which have potential anticancer activity, are often screened out in drug discovery programs for cancer research [1] due to the presence of cells which have the capability to regrow in vivo, called cancer stem cells (CSCs). Thus, the antitumor activity of the compounds in vivo is not adequate for the treatment of cancer in preclinical models. Tumors are maintained by a self-growing CSC population [2]. Research has confirmed the presence of cancer stem cells in leukemia [3], as well as in tumors of the breast [4], brain [5], lung [6] and colon [7]. In cancer relapse, CSCs must have resisted the primary drug action [8]. Literature has reported that aldehyde dehydrogenase 1 (ALDH-1) is a more potent marker of breast CSCs [9,10,11] and ALDH-1–positive cells are resistant to Epirubicin and Paclitaxel [12]. Adult stem cells can be predicted by a side population (SP) phenotype. A SP is confined to the tumorigenic part of the breast cancer cell line MCF-7 [13,14]. Normal chemotherapy could lead to augmentation of CSCs in treated patients [15,16]. Thus, there remains an urgent need to discover new drugs to effectively eliminate both proliferating cells and CSCs in order to treat cancer [17].

**Figure 1:**
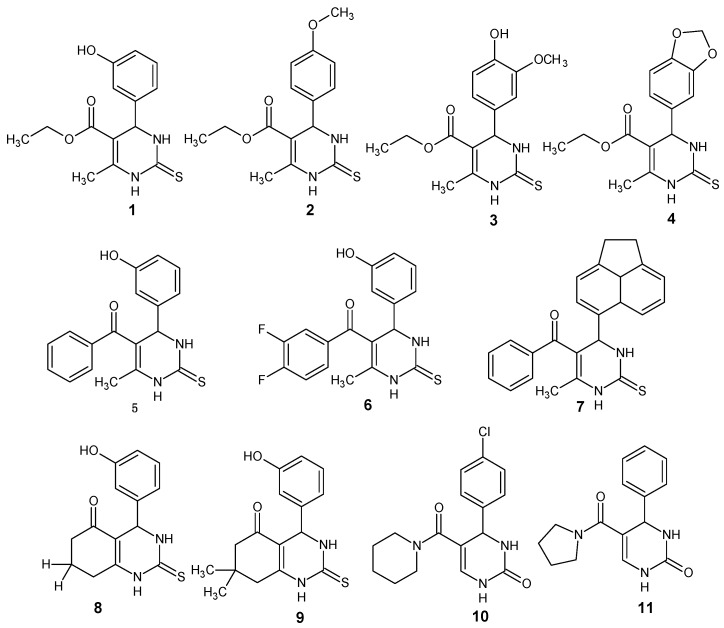
Dihydropyrimidine derivatives demonstrating anticancer activity.

Multicomponent reactions (MCR) are important in the discovery of new lead compounds. The acid-catalyzed cyclocondensation reaction of a diketone with benzaldehyde and urea was reported in 1893 by Pietro Biginelli. The product obtained was identified as a dihydropyridimidine-2-one. Dihydropyrimidines presented a varied range of biological activities, e.g., calcium channel blockers, α-adrenoceptor selective antagonists and anti-mitotics [18]. Furthermore, (*S*)-Monastrol (**1**) has been identified as a novel molecule for the development of potentially new anticancer drugs [19]. Monastrol causes specific and reversible inhibition of kinesin Eg5. Oxo-Monastrol and its thio-analogues have been investigated for their anti-proliferative activity. The 4-methoxy derivative **2** and 3-methoxy-4-hydoxy derivative **3** of Monastrol have been synthesized as anticancer agents [20]. The 3,4-methylenedioxy derivative of Monastrol, Piperastrol (**4**), was found to be three times more potent than Monastrol [21]. Pyrimidinone peptoid hybrids have been reported as active against SKBr-3 breast cancer cells [22]. Improved efficiency was reported in cell-based assays by optimization of the Monastrol-based dihydropyrimidine (DHPM) analogue *R*-Monastrol-97 (**5**) [23]. The 3,4-difluoro derivative *R*-fluorestrol (**6**) was also reported to be a potent anticancer compound. Compound **7**, derived from Monastrol-97, has been reported to be active in anticancer screens. Deaths of over 80% of cancer cells were observed after 72 h of treatment with the Biginelli adducts Enastron (**8**) and dimethyl Enastron (**9**) [24]. These compounds showed minute toxic effects against healthy fibroblast cells. Amide-derived Biginelli adducts exhibited moderate anti-proliferative activity against HepG2 epithelial carcinoma. Compounds **10** and **11** showed IC_50_ values of (190 μg/mL) against HeLa hepatocellular carcinoma cells [25]. Additionally, cinnamoyl derivatives of dihydropyrimidine have been reported as potent anticancer agents [26]. Examples of dihydropyrimidines demonstrating anticancer activities are presented in Figure 1.

**Figure 2:**
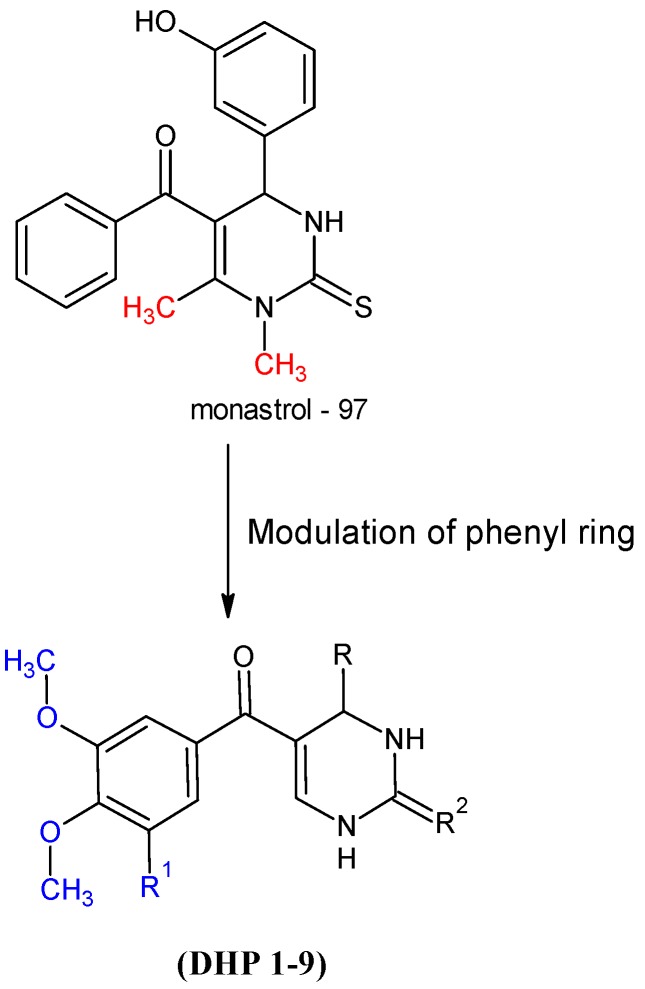
Lead compound Monstrol-97 and newly synthesized compounds (**DHP 1**–**9**).

There is a need for structural optimization of dihydropyrimidine derivatives with the aim of modifying the profile of current lead molecules. In an effort to discover novel dihydropyrimidine derivatives with potent anticancer activity against cancer stem cells, modulation of the Monastrol-97 structure was carried out as illustrated in Figure 2.

These dihydropyrimidine derivatives were then evaluated for antitumor activity.

## 2. Results and Discussion

Enaminones **E1** and **E2** were reacted with substituted benzaldehydes and urea/thiourea in the presence of acetic acid to yield dihydropyrimidinone/thione derivatives (**DHP 1**–**9**). The purity of the compounds was confirmed by elemental analysis and thin-layer chromatography. The compounds were characterized using spectroscopic methods. In the ^1^H-NMR spectra, the signals of the individual protons of the compounds were verified on the basis of multiplicity, chemical shifts and the coupling constant. All the compounds showed the D_2_O exchangeable broad singlet at 8.8–9.8 ppm and 9.5–10.5 ppm corresponding to the two NH protons. Analytical and spectral data for the compounds were in good agreement with the expected structures of the synthesized derivatives. The physicochemical properties of all compounds are given in Table 1.

**Figure 3:**
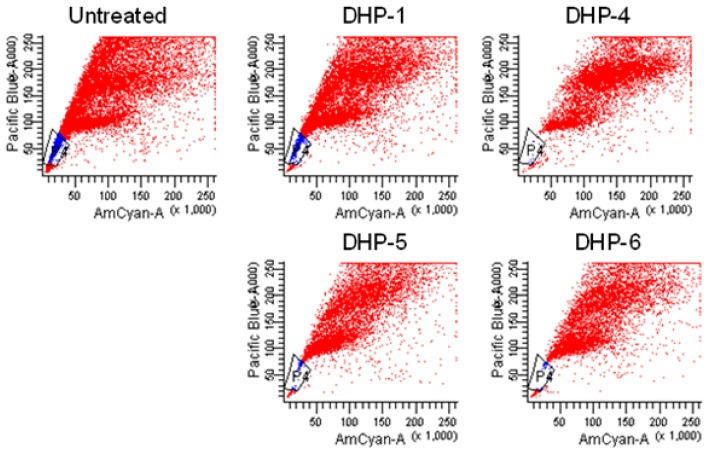
Scatter plot showing results of side population analyses of tumor-derived cells of LOVO untreated, treated with **DHP-1**, **DHP-4**, **DHP-5** and **DHP-6**. Furthermore, compound **DHP-5** exhibit an in vitro anti-proliferative effect and arrested cancer cells at the G2 checkpoint (Figure 4). Blue color represents the percentage of cancer stem cells and red color represents the percentage of remaining cells other than cancer stem cells.

The newly synthesized compounds (**DHP 1**–**9**) were evaluated for side population percent inhibition on colon cancer cell line (LOVO) at a 10 μM concentration (Figure 3, Table 2).

The structure-activity relationships of the compounds were studied. From the compounds (**DHP 1**–**9**), four compounds were found to be very effective, namely **DHP-4**, **DHP-6**, **DHP-2** and **DHP-3**, when the side population inhibition percentage was measured at a 10 μM concentration. Compounds **DHP-5** and **DHP-8** were moderately active as indicated by a low value of the side population inhibition percentage. Most of the dihydropyrimidine compounds (**DHP 1**–**9**) presented significant activity against side population inhibition percentage. It was noted that most of the compounds having a methoxy group at R^1^ were active. Compounds with an oxygen atom at R^2^ were also active. Compound **DHP-6**, with a hydrogen at R^1^ and a sulfur atom at R^2^, displayed significant activity. Compound **DHP-4** was found to be the most active compound of the series.

**Figure 5:**
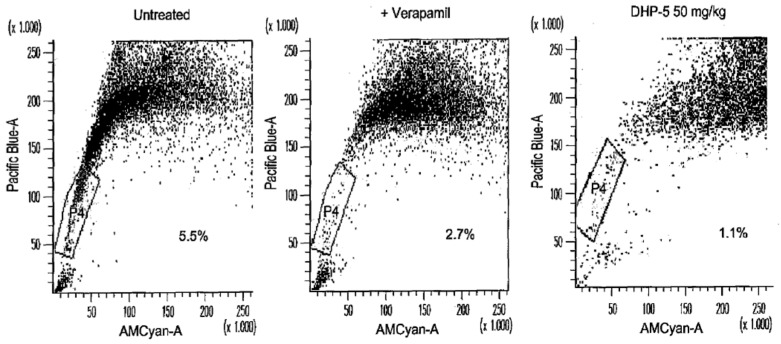
Scatter plot showing results of side population analyses of tumor-derived cells of the LOVO xenograft that were untreated, treated with side population inhibitor reference drug Verapamil (200 μM) and with **DHP-5** (50 μM).

A side population analysis of tumor-derived cells of LOVO xenografts that were untreated, treated with the side population inhibitor reference drug Verapamil 200 μM, and with compound **DHP-5** (50 μM) confirmed that **DHP-5** had a more potent inhibitory effect on the side population cancer stem cells than the reference drug Verapamil (Figure 5).

**Figure 6:**
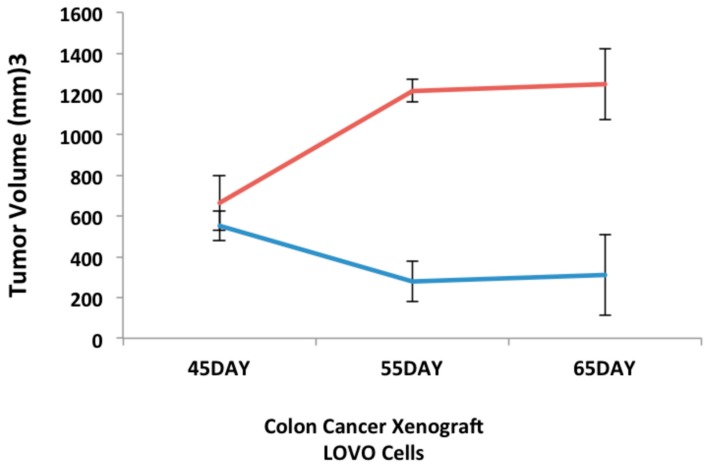
Graph showing tumor growth record of LOVO (colon cancer xenograft) in untreated mice group (**red line**) and **DHP-5**–treated (50 mg/kg) mice group (**blue line**).

The tumor growth of LOVO (colon cancer xenografts) was recorded in untreated mice groups and in **DHP-5**–treated (50 mg/kg) mice groups. A potent anti-tumor effect was demonstrated by a shrinking of tumors in the animals which were treated by compound **DHP-5**. A remarkable anti-tumor effect of compound **DHP-5** was demonstrated on tumors of colon cancer xenografts (Figure 6).

## 3. Material and Methods

### 3.1. Experimental

All solvents were obtained from Merck (Kenilworth, NJ, USA). The homogeneity of the compounds was checked by TLC performed on silica gel G; An iodine chamber was used for visualization of TLC spots. The FT-IR spectra were recorded in KBr pellets on a Spectrum BX Perkin Elmer FT-IR spectrophotometer (Perkin Elmer, Hopkinton, MA, USA). Melting points were determined on a Gallenkamp melting point apparatus (Gallenkamp, Loughborough, UK), and are uncorrected. NMR spectra were scanned in DMSO-*d*_6_ on a Bruker NMR spectrophotometer (Bruker, Billerica, MA, USA) operating at 500 MHz for ^1^H and 125.76 MHz for ^13^C at the Research Center, College of Pharmacy, King Saud University, Saudi Arabia. Chemical shifts δ are expressed in parts per million (ppm) relative to TMS as an internal standard and D_2_O was added to confirm the exchangeable protons. Coupling constants (*J*) are in Hertz. The molecular masses of compounds were determined by UPLC/TQMS and all tested compounds yielded data consistent with a purity of ≥95%, as measured by HPLC (Agilent 1260 affinity). The elemental analyses (C, H, N (±0.4%); and S (±0.3%)) were performed on a CHN Elementar (Analysensysteme GmbH, Langenselbold, Germany).

**Scheme 1:**
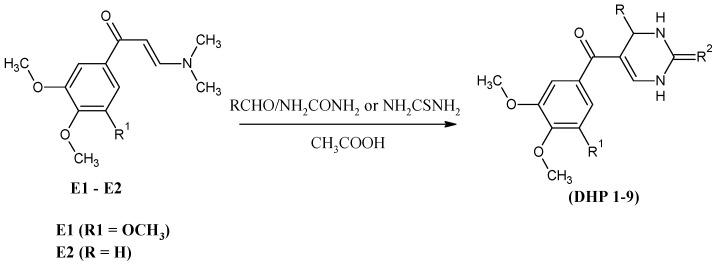
Synthetic route of compounds (**DHP 1**–**9**).

The synthesis of dihydropyrimidine derivatives was carried out in single step as shown in Scheme 1.

### 3.2. General Synthesis of 4-(Substituted phenyl)-5-(3,4,5-trimethoxybenzoyl/3,4-dimethoxybenzoyl)-3,4-dihydropyrimidin-2(1H)-ones ***DHP 1**–**9***

A solution of enaminone **E1/E2** (0.01 mol), substituted benzaldehyde (0.01 mol), urea/thiourea (0.01 mol) and glacial acetic acid (10 mL) was heated under reflux for 3 h. The precipitates (**DHP 1**–**9**) thus formed were collected by filtration, washed with water and recrystallized from acetic acid.

*4-Phenyl-5-(3,4,5-trimethoxybenzoyl)-3,4-dihydropyrimidin-2(1H)-one* (**DPH-1**): Yield: 70%; m.p.: 153–155 °C; IR (KBr): 3412 (N-H), 2938 (ArC-H), 1700 (C=O), 1636 (C=O), 1618 (C=C), 1126 (C-O); ^1^H-NMR (500 MHz, DMSO-*d*_6_); δ = 3.80 (9H, s, 3× -OCH_3_), 5.40 (1H, d, *J* = 2.5 Hz, H-4), 6.73–7.36 (7H, m, Ar-H), 7.88 (1H, d, *J* = 2.5 Hz, =CH), 9.50 (1H, bs, NH, D_2_O exchg.), 10.00 (1H, bs, NH, D_2_O exchg.); ^13^C-NMR (125.76 MHz, DMSO-*d*_6_): δ = 54.0, 56.4, 56.5, 56.7, 60.5, 106.2, 112.4, 126.9, 127.8, 128.9, 134.4, 140.3, 142.3, 144.6, 151.8, 152.9, 153.0, 191.0; MS: *m*/*z* = 368.46 [M]^+^; Analysis: C_20_H_20_N_2_O_5_ for, calcd. C 65.21, H 5.47, N 7.60%; found C 65.45, H 5.48, N 7.62%.

*4-(4-Chlorophenyl)-5-(3,4,5-trimethoxybenzoyl)-3,4-dihydropyrimidin-2(1H)-one* (**DPH-2**): Yield: 75%; m.p.: 138–140 °C; IR (KBr): 3412 (N-H), 2938 (ArC-H), 1686 (C=O), 1654 (C=O), 1618 (C=C), 1123 (C-O); ^1^H-NMR (500 MHz, DMSO-*d*_6_); δ = 3.79 (9H, s, 3× -OCH_3_), 5.39 (1H, d, *J* = 3.0 Hz, H-4), 6.74–7.43 (6H, m, Ar-H), 7.91 (1H, d, *J* = 2.5 Hz, =CH), 9.50 (1H, bs, NH, D_2_O exchg.), 10.00 (1H, bs, NH, D_2_O exchg.); ^13^C-NMR (125.76 MHz, DMSO-*d*_6_): δ = 53.6, 56.4, 56.5, 56.7, 60.5, 60.7, 106.2, 108.2, 112.0, 128.4, 128.9, 128.9, 129.8, 131.6, 152.3, 134.2, 139.8, 140.2, 140.3, 142.5, 143.5, 151.6, 152.9, 153.0, 153.2, 191.0, 192.5, 193.0; MS: *m*/*z* = 402.8 [M]^+^, 403.8 [M + 1]^+^; Analysis: C_20_H_19_N_2_O_5_Cl for, calcd. C 59.63, H 4.75, N 6.95%; found C 59.45, H 4.73, N 6.97%.

*4-(4-Nitrophenyl)-5-(3,4,5-trimethoxybenzoyl)-3,4-dihydropyrimidin-2(1H)-one* (**DPH-3**): Yield: 65%; m.p.: 158–160 °C; IR (KBr): 3421 (N-H), 2936 (ArC-H), 1685 (C=O), 1654 (C=O), 1618 (C=C), 1125 (C-O); ^1^H-NMR (500 MHz, DMSO-*d*_6_); δ = 3.77 (9H, s, 3× -OCH_3_), 5.53 (1H, d, *J* = 2.5 Hz, H-4), 6.74–7.40 (6H, m, Ar-H), 8.20 (1H, d, *J* = 2.5 Hz, =CH), 9.47 (1H, bs, NH, D_2_O exchg.), 10.20 (1H, bs, NH, D_2_O exchg.); ^13^C-NMR (125.76 MHz, DMSO-*d*_6_): δ = 53.8, 56.5, 56.5, 56.7, 60.2, 60.5, 60.7, 65.3, 106.2, 124.7, 128.4, 131.0, 134.1, 138.2, 140.4, 143.0, 147.2, 151.4, 151.7, 153.0, 153.0, 153.2, 190.9; MS: *m*/*z* = 413.47 [M]^+^; Analysis: C_20_H_19_N_3_O_7_ for, calcd. C 58.11, H 4.63, N 10.16%; found C 58.32, H 4.62, N 10.19%.

*4-(3,4-Dimethoxyphenyl)-5-(3,4,5-trimethoxybenzoyl)-3,4-dihydropyrimidin-2(1H)-one* (**DPH-4**): Yield: 72%; m.p.: 165–167 °C; IR (KBr): 3367 (N-H), 2937 (ArC-H), 1700 (C=O), 1624 (C=O), 1578 (C=C), 1123 (C-O); ^1^H-NMR (500 MHz, DMSO-*d*_6_); δ = 3.81 (15H, s, 5× -OCH_3_), 5.36 (1H, d, *J* = 2.5 Hz, H-4), 6.75–7.28 (5H, m, Ar-H), 7.81 (1H, d, *J* = 2.5 Hz, =CH), 9.24 (1H, bs, NH, D_2_O exchg.), 9.84 (1H, bs, NH, D_2_O exchg.); ^13^C-NMR (125.76 MHz, DMSO-*d*_6_): δ = 53.5, 55.9, 56.0, 56.3, 56.4, 56.5, 56.7, 60.5, 60.7, 65.3, 106.1, 106.2, 108.2, 109.9, 111.0, 112.2, 116.4, 118.8, 120.3, 126.5, 130.1, 131.8, 134.4, 135.1, 136.9, 139.3, 140.1, 142.1, 147.6, 148.6, 149.6, 151.7, 152.9, 153.0, 154.6, 193.3; MS: *m*/*z* = 428.26 [M]^+^; Analysis: C_22_H_24_N_2_O_7_ for, calcd. C 61.67, H 5.65, N 6.54%; found C 61.45, H 5.66, N 6.56%.

*4-(4-Ethoxyphenyl)-5-(3,4,5-trimethoxybenzoyl)-3,4-dihydropyrimidin-2(1H)-one* (**DPH-5**): Yield: 60%; m.p.: 168–170 °C; IR (KBr): 3411 (N-H), 2938 (ArC-H), 1696 (C=O), 1648 (C=O), 1618 (C=C), 1126 (C-O); ^1^H-NMR (500 MHz, DMSO-*d*_6_); δ = 1.31 (3H, t, *J* = 7.0 Hz, -CH_3_), 3.80 (9H, s, 3× -OCH_3_), 4.20 (2H, q, *J* = 2.0 Hz, -OCH_2_), 5.32 (1H, d, *J* = 2.5 Hz, H-4), 6.75–7.25 (6H, m, Ar-H), 7.79 (1H, d, *J* = 2.5 Hz, =CH), 8.81 (1H, bs, NH, D_2_O exchg.), 9.50 (1H, bs, NH, D_2_O exchg.); ^13^C-NMR (125.76 MHz, DMSO-*d*_6_): δ = 15.1, 53.3, 56.5, 60.5, 63.4, 106.1, 112.7, 114.7, 128.1, 140.2, 153.0, 192.0; MS: *m*/*z* = 412.28 [M]^+^; Analysis: C_22_H_24_N_2_O_6_ for, calcd. C 64.07, H 5.87, N 6.79%; found C 64.25, H 5.88, N 6.76%.

*(3,4-Dimethoxyphenyl)(4-phenyl-2-thioxo-1,2,3,4-tetrahydropyrimidin-5-yl)methanone* (**DHP-6**): Yield: 65%; m.p.: 248–250 °C; IR (KBr): 3413 (N-H), 2955 (ArC-H), 1653 (C=O), 1636 (C=O), 1595 (C=C), 1199 (C-O); ^1^H-NMR (500 MHz, DMSO-*d*_6_); δ = 3.81 (6H, s, 2× -OCH_3_), 5.45 (1H, d, *J* = 3.0 Hz, H-4), 6.97–7.28 (7H, m, Ar-H), 7.34 (1H, d, *J* = 3.0 Hz, =CH), 9.70 (1H, bs, NH, D_2_O exchg.), 10.40 (1H, bs, NH, D_2_O exchg.); ^13^C-NMR (125.76 MHz, DMSO-*d*_6_): δ = 54.2, 56.6, 56.1, 56.3, 111.1, 111.2, 111.7, 112.2, 113.6, 122.7, 127.1, 128.2, 129.1, 130.8, 136.7, 143.4, 149.1, 149.3, 152.2, 153.8, 162.7, 174.3, 191.0, 193.5; MS: *m*/*z* = 355.0 [M + 1]^+^; Analysis: C_19_H_18_N_2_O_3_S for, calcd. C 64.39, H 5.12, N 7.90, S 9.05%; found C 64.54, H 5.11, N 7.92, S 9.04%.

*(3,4-Dimethoxyphenyl)(4-chlorophenyl-2-thioxo-1,2,3,4-tetrahydropyrimidin-5-yl)methanone* (**DHP-7**): Yield: 65%; m.p.: 243–245 °C; IR (KBr): 3413 (N-H), 2933 (ArC-H), 1670 (C=O), 1647 (C=O), 1616 (C=C), 1195 (C-O); ^1^H-NMR (500 MHz, DMSO-*d*_6_); δ = 3.78 (6H, s, 2× -OCH_3_), 5.45 (1H, d, *J* = 3.0 Hz, H-4), 6.98–7.45 (7H, m, Ar-H), 7.96 (1H, d, *J* = 3.0 Hz, =CH), 9.76 (1H, bs, NH, D_2_O exchg.), 10.49 (1H, bs, NH, D_2_O exchg.); ^13^C-NMR (125.76 MHz, DMSO-*d*_6_): δ = 53.7, 56.0, 56.1, 111.1, 111.7, 113.2, 122.7, 129.0, 129.1, 130.7, 132.8, 137.0, 142.3, 149.1, 152.2, 162.7, 174.3, 190.9; MS: *m*/*z* = 387.99 [M]^+^; Analysis: C_19_H_17_N_2_O_3_ClS for, calcd. C 58.68, H 4.41, N 7.20, S 8.25%; found C 58.85, H 4.43, N 7.23, S 8.24%.

*(3,4-Dimethoxyphenyl)(4-nitrophenyl-2-thioxo-1,2,3,4-tetrahydropyrimidin-5-yl)methanone* (**DHP-8**): Yield: 68%; m.p.: 258–260 °C; IR (KBr): 3412 (N-H), 2933 (ArC-H), 1676 (C=O), 1654 (C=O), 1615 (C=C), 1141 (C-O); ^1^H-NMR (500 MHz, DMSO-*d*_6_); δ = 3.82 (6H, s, 2× -OCH_3_), 5.58 (1H, d, *J* = 3.0 Hz, H-4), 7.0–7.95 (7H, m, Ar-H), 8.26 (1H, d, *J* = 2.5 Hz, =CH), 9.85 (1H, bs, NH, D_2_O exchg.), 10.59 (1H, bs, NH, D_2_O exchg.); ^13^C-NMR (125.76 MHz, DMSO-*d*_6_): δ = 53.9, 56.0, 56.1, 111.1, 111.7, 112.6, 122.8, 124.4, 128.5, 130.6, 137.5, 147.4, 149.1, 150.3, 152.3, 162.7, 174.5, 190.8; MS: *m*/*z* = 402.23 [M + 3]^+^; Analysis: C_19_H_17_N_3_O_5_S for, calcd. C 57.13, H 4.29, N 10.52, S 8.03%; found C 57.23, H 4.28, N 10.55, S 8.01%.

*(3,4-Dimethoxyphenyl)(3,4-dimethoxyphenyl-2-thioxo-1,2,3,4-tetrahydropyrimidin-5-yl)methanone* (**DHP-9**): Yield: 70%; m.p.: 228–230 °C; IR (KBr): 3410 (N-H), 2932 (ArC-H), 1684 (C=O), 1654 (C=O), 1611 (C=C), 1134 (C-O); ^1^H-NMR (500 MHz, DMSO-*d*_6_); δ = 3.81 (12H, s, 4× -OCH_3_), 5.40 (1H, d, *J* = 3.0 Hz, H-4), 6.82–7.21 (6H, m, Ar-H), 7.96 (1H, d, *J* = 2.5 Hz, =CH), 9.68 (1H, bs, NH, D_2_O exchg.), 10.38 (1H, bs, NH, D_2_O exchg.); ^13^C-NMR (125.76 MHz, DMSO-*d*_6_): δ = 43.7, 55.9, 56.0, 56.0, 56.1, 111.1, 111.7, 112.3, 113.5, 119.0, 122.7, 130.8, 135.7, 136.6, 148.9, 149.1, 149.1, 152.2, 162.7, 174.1, 191.0; MS: *m*/*z* = 413.6 [M − 1]^+^; Analysis: C_21_H_22_N_2_O_5_S for, calcd. C 60.85, H 5.35, N 6.76, S 7.74%; found C 61.05, H 5.36, N 6.78, S 7.73%.

### 3.3. Cell line and Tissue Culture

LOVO colon cancer cells were purchased from the American Type Culture Collection. LOVO cells were cultured in RPMI. The medium was supplemented with 10% FBS (Cambrex Bio Science, Franklin Lakes, NJ, USA), 100 IU/mL of Penicillin and 100 mg/mL of Streptomycin. Cell viability was assessed by trypan blue exclusion analysis. Cell numbers were determined by using a hemacytometer.

### 3.4. Flow Cytometric Analysis of Cellular DNA Content

Cells (2 × 10^6^) were fixed in 1 mL of ethanol (70%) for 60 min at room temperature. Harvested cells were resuspended in 1 mL of sodium citrate (50 mM) containing 250 μg RNase A and incubated at 50 °C for 60 min Next, cells were resuspended in the same buffer containing 4 μg of propidium iodide (PI) and incubated for 30 min before being analyzed by flow cytometry (Becton Dickinson, San Jose, CA, USA). The percentage of cells in various cell cycle phases was determined by using Cell Quest Pro software (version 5.1, Becton Dickinson, East Rutherford, NJ, USA).

### 3.5. Side Population Staining by DYECYCLE Violet Stain

For DCV staining, cells were pelleted and suspended in DMEM cell culture medium at a concentration of 1 × 10^6^ cells/mL. DCV (Invitrogen Molecular Probes^®^, Eugene, OR, USA) was added at a final staining concentration of 10 μM, as this concentration gave optimal separation between SP and non SP cells. PI staining was used to exclude dead cells. Functionally, to gate only side population cells, Verapamil 200 μM or Emtricitabine (FTC, 10 μg/mL) was used. All analyses were performed on a FACS LSRII (BD Biosciences, San Jose, CA, USA). Debris and cell clusters were excluded during side-scatter and forward-scatter analyses.

### 3.6. Antitumor Activity in Mice

Nude mice (Jackson Laboratories, Bar Harbor, ME, USA), six to seven weeks old, weighing 20 g, were obtained from the Animal Care and Use Committee of the King Faisal Specialist Hospital and Research Centre, Riyadh, KSA. All of the animals were acclimatized to laboratory conditions for one week before experiments. The animals were maintained under standard conditions, housed in a pathogen-free environment, and fed adequately. Each treatment and vehicle group consisted of six animals. The breeding, care and sacrifice of the animals were performed in accordance with the protocols approved by the Animal Care and Use Committee of the King Faisal Specialist Hospital and Research Centre. The mice were injected with 4 × 10^6^ cells of LOVO subcutaneously in the right flank, and tumor size was measured weekly using a caliper. When the tumor reached approximately 400 mm^3^ diameter, the mice were divided into control, treated groups, the treatment including administration of **DHP-5** (50 mg/kg) via intraperitoneal injection daily for 14 days. The general toxicity of the treatment was determined by measuring the total body weight of the treated and control mice.

## 4. Conclusions

In conclusion, we focused on the synthesis of dihydropyrimidine derivatives (**DHP 1**–**9**). The synthesized compounds were screened in vitro against LOVO colon cancer cells. **DHP-4** was found to be the most active compound of the series in its side population inhibition percentage at 10 μM. The anti-tumor effect of compound **DHP-5** was demonstrated on tumors of colon cancer xenografts. Compound **DHP-5** was found to be a more potent inhibitor of side population cells than the reference drug Verapamil. Compound **DHP-5** exhibited an in vitro anti-proliferative effect and arrested cancer cells at the G2 checkpoint. Furthermore, treatment with compound **DHP-5** enabled blocking of the self-renewal ability of breast cancer cells in a dose-dependent manner. Compound **DHP-5** induced apoptosis and blocked cell proliferation in vitro and presented superior efficacy compared to the reference drug Doxorubicin in advanced animal models of colon cancer without any sign of general toxicity.

## Figures and Tables

**Figure 4:**
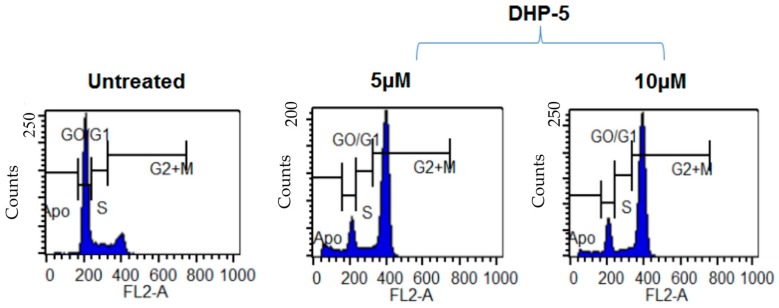
Compound **DHP-5** arrested cancer cells at G2 checkpoint.

**Table 1:**
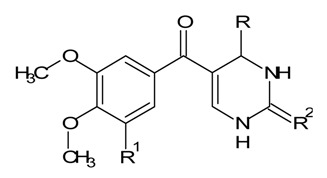
Physical data of the synthesized dihydropyrimidinone/thione compounds (**DHP 1**–**9**).

Compounds	R	R^1^	R^2^	(Yield %)	m.p. (°C)
**DHP-1**	Phenyl	OCH_3_	O	70	153–155
**DHP–2**	4-Chlorophenyl	OCH_3_	O	75	138–140
**DHP-3**	4-Nitrophenyl	OCH_3_	O	65	158–160
**DHP-4**	3,4-Dimethoxyphenyl	OCH_3_	O	72	165–167
**DHP-5**	4-Ethoxyphenyl	OCH_3_	O	60	168–170
**DHP-6**	Phenyl	H	S	65	248–250
**DHP-7**	4-Chlorophenyl	H	S	65	243–245
**DHP-8**	4-Nitrophenyl	H	S	68	258–260
**DHP-9**	3,4-Dimethoxyphenyl	H	S	70	228–230

**Table 2:** Side population inhibition on LOVO colon cancer cells (%) at 10 μM concentration.

Compounds	* Side Population (%) at 10 μM	^#^ Side Population Inhibition (%) at 10 μM
**DHP-1**	4.90 ± 0.2	0
**DHP-2**	1.72 ± 0.1	64.7
**DHP-3**	1.76 ± 0.3	64
**DHP-4**	1.44 ± 0.5	70.5
**DHP-5**	2.01 ± 0.7	58.82
**DHP-6**	1.47 ± 0.6	70
**DHP-7**	4.90 ± 0.3	0
**DHP-8**	2.4 ± 0.1	50
**DHP-9**	4.90 ± 0.1	0

* Side population% as mean ± SD of three independent experiments; ^#^ Inhibition% = 100 − (SP% of treated cells/SP% of untreated cells) × 100.

## References

[1] Winquist R.J., Furey B.F., Boucher D.M. (2010). Cancer stem cells as the relevant biomass for drug discovery. Curr. Opin. Pharmacol..

[2] McDermott S.P., Wicha M.S. (2010). Targeting breast cancer stem cells. Mol. Oncol..

[3] Bonnet D., Dick J.E. (1997). Human acute myeloid leukemia is organized as a hierarchy that originates from a primitive hematopoietic cell. Nat. Med..

[4] Al-Hajj M., Wicha M.S., Benito-Hernandez A., Morrison S.J., Clarke M.F. (2003). Prospective identification of tumorigenic breast cancer cells. Proc. Natl. Acad. Sci. USA.

[5] Singh S.K., Clarke I.D., Terasaki M., Bonn V.E., Hawkins C., Squire J., Dirks P.B. (2003). Identification of a cancer stem cell in human brain tumors. Cancer Res..

[6] Ho M.M., Ng A.V., Lam S., Hung J.Y. (2007). Side population in human lung cancer cell lines and tumors is enriched with stem-like cancer cells. Cancer Res..

[7] Ricci-Vitiani L., Lombardi D.G., Pilozzi E., Biffoni M., Todaro M., Peschle C., de Maria R. (2007). Identification and expansion of human colon-cancer-initiating cells. Nature.

[8] Bomken S., Fiser K., Heidenreich O., Vormoor J. (2010). Understanding the cancer stem cell. Br. J. Cancer.

[9] Ginestier C., Hur M.H., Charafe-Jauffret E., Monville F., Dutcher J., Brown M., Jacquemier J., Viens P., Kleer C.G., Liu S. (2007). ALDH1 is a marker of normal and malignant human mammary stem cells and a predictor of poor clinical outcome. Cell Stem Cell.

[10] Morimoto K., Kim S.J., Tanei T., Shimazu K., Tanji Y., Taguchi T., Tamaki Y., Terada N., Noguchi S. (2009). Stem cell marker aldehyde dehydrogenase 1-positive breast cancers are characterized by negative estrogen receptor, positive human epidermal growth factor receptor type 2, and high Ki67 expression. Cancer Sci..

[11] Charafe-Jauffret E., Ginestier C., Iovino F., Tarpin C., Diebel M., Esterni B., Houvenaeghel G., Extra J.M., Bertucci F., Jacquemier J. (2010). Aldehyde dehydrogenase 1-positive cancer stem cells mediate metastasis and poor clinical outcome in inflammatory breast cancer. Clin. Cancer Res..

[12] Tanei T., Morimoto K., Shimazu K., Kim S.J., Tanji Y., Taguchi T., Tamaki Y., Noguchi S. (2009). Association of breast cancer stem cells identified by aldehyde dehydrogenase 1 expression with resistance to sequential Paclitaxel and epirubicin-based chemotherapy for breast cancers. Clin. Cancer Res..

[13] Kondo T., Setoguchi T., Taga T. (2004). Persistence of a small subpopulation of cancer stem-like cells in the C6 glioma cell line. Proc. Natl. Acad. Sci. USA.

[14] Patrawala L., Calhoun T., Schneider-Broussard R., Zhou J., Claypool K., Tang D.G. (2005). Side population is enriched in tumorigenic, stem-like cancer cells, whereas ABCG2^+^ and ABCG2^−^ cancer cells are similarly tumorigenic. Cancer Res..

[15] Li X., Lewis M.T., Huang J., Gutierrez C., Osborne C.K., Wu M.F., Hilsenbeck S.G., Pavlick A., Zhang X., Chamness G.C. (2008). Intrinsic resistance of tumorigenic breast cancer cells to chemotherapy. J. Natl. Cancer Inst..

[16] Yu F., Yao H., Zhu P., Zhang X., Pan Q., Gong C., Huang Y., Hu X., Su F., Lieberman J. (2007). let-7 regulates self renewal and tumorigenicity of breast cancer cells. Cell.

[17] Al-Hajj M., Becker M.W., Wicha M., Weissman I., Clarke M.F. (2004). Therapeutic implications of cancer stem cells. Curr. Opin. Genet. Dev..

[18] Bokaeva S.S. (1967). The effects of some pyrimidine derivates on the growth of transplanted tumors in animals. Tr. Kaz. Nauchno-Issled. Inst. Onkol. Radiol..

[19] Mayer T.U., Kapoor T.M., Haggarty S.J., King R.W., Schreiber S.L., Mitchison T.J. (1999). Small molecule inhibitor of mitotic spindle bipolarity identified in a phenotype-based screen. Science.

[20] Russowsky D., Canto R.F., Sanches S.A., D’Oca M.G., de Fátima A., Pilli R.A., Kohn L.K., Antônio M.A., de Carvalho J.E. (2006). Synthesis and differential antiproliferative activity of Biginelli compounds against cancer cell lines: Monastrol, oxo-monastrol and oxygenated analogues. Bioorg. Chem..

[21] Prokopcova H., Dallinger D., Uray G., Kaan H.Y., Ulaganathan V., Kozielski F., Laggner C., Kappe C.O. (2010). Structure-activity relationships and molecular docking of novel dihydropyrimidine-based mitotic Eg5 inhibitors. ChemMedChem.

[22] Wright C.M., Chovatiya R.J., Jameson N.E., Turner D.M., Zhu G., Werner S., Huryn D.M., Pipas J.M., Day B.W., Wipf P. (2008). Pyrimidinone-peptoid hybrid molecules with distinct effects on molecular chaperone function and cell proliferation. Bioorg. Med. Chem..

[23] Garcia-Saez I., DeBonis S., Lopez R., Trucco F., Rousseau B., Thuéry P., Kozielski F. (2007). Structure of human Eg5 in complex with a new monastrol-based inhibitor bound in the R configuration. J. Biol. Chem..

[24] Ramos L.M., Guido B.C., Nobrega C.C., Corrêa J.R., Silva R.G., de Oliveira H.C., Gomes A.F., Gozzo F.C., Neto B.A. (2013). The biginelli reaction with an imidazolium-tagged recyclable iron catalyst: Kinetics, mechanism, and antitumoral activity. Chem. Eur. J..

[25] Soumyanarayanan U., Bhat V.G., Kar S.S., Mathew J.A. (2012). Monastrol mimic Biginelli dihydropyrimidinone derivatives: Synthesis, cytotoxicity screening against HepG2 and HeLa cell lines and molecular modeling study. Org. Med. Chem. Lett..

[26] Kumar B.R.P., Sankar G., Nasir Baig R.B., Chandrashekaran S. (2009). Novel Biginelli dihydropyrimidines with potential anticancer activity: A parallel synthesis and CoMSIA study. Eur. J. Med. Chem..

